# Children and Adolescents’ Ingroup Biases and Developmental Differences in Evaluations of Peers Who Misinform

**DOI:** 10.3389/fpsyg.2022.835695

**Published:** 2022-04-15

**Authors:** Aqsa Farooq, Eirini Ketzitzidou Argyri, Anna Adlam, Adam Rutland

**Affiliations:** Department of Psychology, University of Exeter, Exeter, United Kingdom

**Keywords:** misinformation, moral development, children, adolescents, intergroup, intentionality attribution

## Abstract

Previous developmental research shows that young children display a preference for ingroup members when it comes to who they accept information from – even when that information is false. However, it is not clear how this ingroup bias develops into adolescence, and how it affects responses about peers who misinform in intergroup contexts, which is important to explore with growing numbers of young people on online platforms. Given that the developmental span from childhood to adolescence is when social groups and group norms are particularly important, the present study took a Social Reasoning Developmental Approach. This study explored whether children and adolescents respond differently to a misinformer spreading false claims about a peer breaking COVID-19 rules, depending on (a) the group membership of the misinformer and their target and (b) whether the ingroup had a “critical” norm that values questioning information before believing it. 354 United Kingdom-based children (8–11 years old) and adolescents (12–16 years old) read about an intergroup scenario in which a peer spreads misinformation on WhatsApp about a competitor. Participants first made moral evaluations, which asked them to judge and decide whether or not to include the misinformer, with follow-up “Why?” questions to capture their reasoning. This was followed by asking them to attribute intentions to the misinformer. Results showed that ingroup preferences emerged both when participants morally evaluated the misinformer, and when they justified those responses. Participants were more likely to evaluate an ingroup compared to an outgroup misinformer positively, and more likely to accuse an outgroup misinformer of dishonesty. Adolescents attributed more positive intentions to the misinformer compared with children, with children more likely to believe an outgroup misinformer was deliberately misinforming. The critical norm condition resulted in children making more positive intentionality attributions toward an ingroup misinformer, but not an outgroup misinformer. This study’s findings highlight the importance of shared group identity with a misinformer when morally evaluating and reasoning about their actions, and the key role age plays in intentionality attributions surrounding a misinformer when their intentions are ambiguous.

## Introduction

Misinformation is false information which circulates as the truth and has been regarded as one of modern society’s biggest threats (Lewandowsky et al., 2017), and yet on certain popular social media platforms, it is more widespread than real news (Shin et al., 2018). This is a particular concern given that reports show that 55% of 12–15 years old get their news from social media (Office of Communications, 2020). According to the adult literature, one of the leading causes of belief in and spreading of misinformation is the desire to sustain and propagate the views held by one’s social (e.g., political) group, regardless of accuracy, often to maintain the acceptance within that group (Levy et al., 2021). Understanding whether a similar dynamic occurs in childhood, and when, may inform ways to tackle the spread of misinformation when it originates from identification with and loyalty toward the source (i.e., social group), rather than accuracy. The present study aimed to investigate for the first time, development differences in how individuals spreading misinformation (misinformers) are evaluated depending on the group peership of the misinformer and their target, as well as the children’s or adolescents’ group norms.

### Social Reasoning Developmental Approach

Spreading misinformation can be perceived as a moral transgression, yet we know it often emerges to serve the concerns of social groups (i.e., sustain their beliefs). This suggests both the development of morality and intergroup processes are key to understanding if and when children and adolescents accept misinformers. The present study, therefore, took a Social Reasoning Developmental (SRD; Rutland et al., 2010) approach to children’s evaluations of misinformers in intergroup contexts, since this theory emphasizes both the role of moral and intergroup process (e.g., group identity and group norms) in children’s social and moral decision making. The SRD approach draws from Social Identity Theory (SIT; Tajfel and Turner, 1986) and Social Domain Theory (SDT: Turiel, 1998; Smetana, 2013). SIT contends that individuals value social groups they share identities with (e.g., gender and age) and as a result, are motivated to favor peers who are ingroup peers. It is therefore expected that children’s ingroup favoritism will also be evident in evaluations of individuals who misinform about ingroup or outgroup peers. Research drawing from the SDT approach shows there are three different domains of knowledge from which children draw from when evaluating social and moral events: the moral domain (i.e., concerns about welfare, fairness and deception), the social-conventional domain (i.e., concerns about group functioning, group norms and group identity) and the psychological domain (i.e., an individual’s mental states, preferences, traits and autonomy).

Previous research using the SRD approach has documented that when it comes to evaluating peers who commit acts with moral and social implications, such as group-based exclusion, children tend to focus on concerns about morality, e.g., whether it is right or wrong to do so (Killen et al., 2013). However, as children get older and enter adolescence they begin also to pay attention to social-conventional matters, e.g., what does it mean for their group (Rutland et al., 2015). From late childhood into adolescence we typically see reasoning drawing from a wider range of domains, including the social-conventional or even the psychological domain, e.g., whether it is their personal choice or perspective (Killen and Rutland, 2011). It was therefore expected, in the present study, that children will mostly refer to moral concerns, whereas adolescents will also cite other domains, such as social-conventional or psychological, when evaluating a misinformer in an intergroup context. To our knowledge there has not been any research using the SRD approach to investigating how children and adolescents evaluate a misinformer.

### Children’s Acceptance of Information

A big part of living in our current digital society involves being presented with information from various sources and with varying levels of accuracy. It is, therefore, vital to understand how young, developing minds determine who to accept information from, and when the information source, or who the information is about, matters more than its accuracy. Past research shows that young children up to the age of 7 years old prefer new information that comes from ingroup peers rather than outgroup peers (Chen et al., 2013) and their acceptance of information about an outgroup peer is higher when the source of the information is an ingroup peer (Aldan and Soley, 2019). Alarmingly, this ingroup bias persists even if the information is false. In children as young as 4 years old, inaccurate testimonies about the placement of a toy are accepted when it comes from an ingroup peer over an outgroup peer (McDonald and Ma, 2016). What remains unclear is how this ingroup bias, which arguably makes children more susceptible to believing false information, impacts their moral judgments about the source of the false information. This is particularly important to know from a moral development perspective, as the act of giving out false information, if done with the intention to deceive, can be regarded as having moral implications (Evans and Lee, 2013).

### Children’s Judgments of Morality and Intentionality

Traditionally, children’s moral evaluations have been measured through their assignment of punishment and decisions about whether to include or exclude someone (Killen and Rutland, 2011) and typically, children strive for fairness and equal treatment. However, when evaluating morally dubious behavior such as not sharing resources equally, ingroup biases become prevalent in children’s moral judgments. For example, when children aged 6 and 8 years old were tasked with assigning punishments for selfish behavior, ingroup favoritism and outgroup biases emerged, resulting in harsher punishments for outgroup peers committing the same selfish transgression as their ingroup counterparts (Jordan et al., 2014). These findings indicate that even amongst children who generally prefer to be fair, witnessing morally inappropriate behavior can elicit intergroup biases that can ultimately influence moral evaluations of ingroup and outgroup peers. This suggests that the group peership of the individual committing the immoral act is an important indicator of how the children will evaluate it and was expected to be an important determinant of how children and adolescents evaluated a misinformer in the present study.

Research also suggests that the intentions of the person committing the morally dubious act are important when children make social and moral judgments (Killen et al., 2011). This is key in the context of misinformation in particular, as the act of sharing misinformation can be perceived differently depending on whether the sharer’s intent has been regarded as deliberate or accidental. For children, perceiving someone as deliberately or accidentally sharing misinformation, requires a level of mental state understanding and ability to infer intentionality (Perner, 1997). This ability, however, is subjected to developmental differences. According to research, from the age of 5 years old, children start to consider the intentions of a character when making social and moral judgments about their lying behavior, however, they tend to struggle to tell the difference between intentionally and unintentionally deceptive statements (Peterson, 1995). As a result, when young children attribute intentions to a supposed moral transgressor, they tend to make more negative evaluations than older children. For instance, younger children are more likely to assume the transgressor deliberately engaged in the transgression, whereas older children apply their more advanced perspective taking skills to consider the transgressor’s point of view. So, the attribution of intentions increases in positivity as children get older (Killen et al., 2011). This trend continues into middle childhood up to the age of 11 years (Jambon and Smetana, 2013).

In addition to these developmental differences, group peership and consequently ingroup biases can also influence intentionality evaluations. For instance, from as young as 5 years of age, children refer to an ingroup peer’s mental state more than an outgroup peer’s (McLoughlin and Over, 2017) and older children make more accurate inferences about mental states for similar ingroup peers than outgroup peers (Gönültaş et al., 2020). This suggests that even measurements of intentionality are susceptible to ingroup biases, and so can potentially result in children making mental state assessments about individuals that are first and foremost based on their group peership. Therefore, we would expect that children’s and adolescents’ intentionality attributions of the misinformer will be related to the group membership of the misinformer. Specifically, we anticipate that the attributions will be more positive when there is an ingroup misinformer/outgroup victim compared to an outgroup misinformer/ingroup victim.

What remains unclear is whether these ingroup biases come into effect in the context of morally evaluating someone who is spreading false information with intentions that are ambiguous. If they do so, it is also unknown how these evaluations develop over the course of late childhood and adolescence, when attribution of intention can become more positive (Killen et al., 2011), but is also when exposure to information from misleading and deceptive sources increases, with growing use of social media (Office of Communications, 2020). This is of particular interest given the influence of group norms also start to become prevalent from late childhood onward (Abrams and Rutland, 2012) and can influence how children and adolescents morally judge someone who is spreading misinformation.

### The Importance of Group Norms and Group Loyalty

With age, children start paying increased attention to their group’s norm, and even make moral judgments, such as whether or not to exclude someone, based upon the norms of their ingroup (Hitti et al., 2014). Similarly, due to the importance of groups, and how central they can be to one’s social identity (as per SIT; Tajfel and Turner, 1986), loyalty to the group is a key expectation of group peership, and loyal ingroup peers are typically preferred from late childhood onward (Abrams and Rutland, 2012). According to developmental research, from middle childhood, the importance of showing loyalty to one’s group norm is regarded as a way of staying included and accepted in it (Killen et al., 2013; Rutland et al., 2015). It is, therefore, of interest to explore whether group peers default to showing loyalty to their group, even when not explicitly told to do so, and even when their group peers commit potentially immoral acts such as spreading misinformation about an outgroup peer and so disadvantaging the outgroup. This can have important implications for addressing ways in which belief in misinformation, and support for people who spread misinformation, can be tackled.

### Critical Thinking Against Susceptibility to Misinformation

One way to tackle the spread of misinformation amongst children is by introducing them to the idea of critical thinking. This would promote a way of thinking that encourages being questioning and evaluative about information and its source. While research shows that adolescents and young adults are relatively poor at discerning false information from credible news, there is promising evidence to suggest that improved critical thinking skills can lead to better ability to identify misinformation (Kahne and Bowyer, 2017; Nygren and Guath, 2018). This suggests that questioning and investigative skills, which encourages striving for accuracy rather than solely following group-based beliefs, may help children and adolescents overcome the ingroup biases that lead them to support false testimonies. Individuals can develop such critical thinking, but these skills can also be perceived as normative (i.e., expected) for a social group and become key to how they define their group. It is important to investigate whether creating an ingroup norm that promotes being critical about information can override ingroup biases amongst children and adolescents when morally evaluating a misinformer.

### The Present Study

The aim of the present study was to explore the factors that influence children’s (8–11-year-old) and adolescents’ (12–16-year-old) moral evaluations and intentionality attributions with regards to a peer spreading misinformation about another peer within an intergroup context. These age groups were chosen for a number of reasons. Firstly, around 8 years old is when children become capable of nuanced reasoning that considers factors pertaining to others’ beliefs and mental states, as well as social and moral concerns, simultaneously (Wainryb et al., 1998). As a result, children younger than 7–8 years old understand experiences relating to psychological harm (e.g., name-calling) differently to older children (Helwig et al., 1995), which could extraneously influence understanding of the present study’s context. Furthermore, a recent report shows that 21% of United Kingdom children aged 8–11 years who go online have a social media profile, and this significantly increases to 71% of 12–15-year-old (Office of Communications, 2020). For the purpose of interventions, we wanted to understand how these age groups in particular differ, given their supposed different level of exposure to online sources, as well as when implementation of interventions would be most effective.

Moral evaluations were measured in the form of making a judgment about the misinformer, as well as the decision to include the misinformer. Intentionality attribution was measured by asking participants to rate the extent to which they thought the misinformer was deliberately spreading misinformation, and so the intentions of the misinformer were unknown to the participants, to capture differences in attributions based on the manipulated factors.

The first factor which was manipulated was the type of ingroup norm participants were prescribed upon being introduced to their school group, which served as the intergroup context for the present study. Half of the participants were assigned to a control condition where the only expectation was to be competitive, and where a default of showing loyalty to fellow ingroup peers was expected given the sample was all above 7 years of age, when children are known to evaluate peers based on their loyalty to the group (Abrams and Rutland, 2012). Indeed, previous research shows that loyalty to the group is a feature of group peership that children understand from approximately 7 years of age (Abrams et al., 2003, 2008). The other half of the participants were assigned to a “critical” ingroup norm condition where there was an explicit norm encouraging group peers to be critical in how they considered information and seek truth above all else. This was to investigate whether being placed in a group that values being critical about information can influence evaluations of someone spreading false information, even if they are from their own group. This ingroup norm is based on the findings about how to counter misinformation which states that critical thinking about the source and accuracy of information is key to being able to detect false or unreliable information (Kahne and Bowyer, 2017; Swire and Ecker, 2018).

The second factor which was manipulated was the group membership of the misinformer and the target of their misinformation. Half of the participants were exposed to an ingroup misinformer who spread misinformation about an outgroup peer, and the other half read about an outgroup misinformer who spread misinformation about a peer of the participant’s own group. This was done to examine whether ingroup biases are present when children and adolescents make moral judgments and attribute the intentionality of someone who is sharing information that is potentially false.

It was therefore expected that both moral evaluations and intentionality attributions would differ depending on both manipulated factors. Due to the past research which has shown children’s intentionality attribution toward a moral transgressor becomes more positive with age (Killen et al., 2011; Jambon and Smetana, 2013; we expected a similar trend to emerge in our sample. Due to the literature that suggests children’s social-moral reasoning about moral transgressors tends to be more concerned with moral factors (Killen et al., 2013) while adolescents’ draws from different domains such as social-conventional or psychological (Rutland et al., 2015), we predicted that the same would occur in the present study’s context.

### Hypotheses

H1: Children and adolescents’ moral evaluations and intentionality attributions of the misinformer were expected to be more positive when they were assigned an ingroup misinformer/outgroup target than an outgroup misinformer/ingroup target.
H2: Children and adolescents’ moral evaluations of the misinformer were expected to be less positive if they were assigned the critical norm condition, compared to if they were assigned to the control condition.
H3: Adolescents were expected to attribute more positive intentions to the misinformer compared with children.
H4: When justifying their moral evaluations of the misinformer, children were expected to be more concerned with moral factors in their reasoning, whereas adolescents were expected to also be concerned with social-conventional or psychological factors in their reasoning.

## Materials and Methods

### Participants

Participants (*N* = 354) were recruited from schools in the South-West of England. The participants consisted of 206 (113 male, 93 female) children 8–11 years old (*M*_*age*_ = 9.40, *SD* = 0.90) and 148 (71 male, 77 female) adolescents 12–16 years old (*M*_*age*_ = 14.16, *SD* = 1.07). This sample size was determined by conducting an *a priori* power analysis for an ANOVA with eight groups under the assumption that there would be main effects and interaction effects in G*Power using an alpha of 0.05, a power of 0.95 and a medium effect size (*η^2^* = 0.25) (Faul et al., 2007). This calculation estimated a required sample size of 210. The sample, which was representative of the non-diverse areas of South-West England where the data were collected, consisted of approximately 66.1% White British, 15.6% White European, 6.8% Dual Heritage, and 8.5% other ethnic backgrounds (including Black, Indian, and Bangladeshi). 3% of participants withheld ethnic identity information. Parental consent and participants’ confirmation to participate was obtained for the whole sample.

### Design

This study used a 2 (age group: children vs. adolescents) × 2 (ingroup norm: control condition vs. critical norm condition) × 2 (group membership: ingroup misinformer/outgroup target vs. outgroup misinformer/ingroup target) between-participants design.

### Procedure

Ethical approval for this study, its procedure and its measures was obtained from the first author’s University. This study only consisted of participants whose parent or guardian had given consent for their child to take part. Participants who were happy to begin the questionnaire were informed about a nationwide inter-school “Spelling Bee” competition, in which their school was taking part, and had made it to the final where they would be competing with a (fictional) rival school from their local area for a much coveted trophy, a picture of which they were shown. Information about the competition detailed how the winner would be decided based on a points system, and that COVID-19 guidelines (which followed official United Kingdom Government COVID-19 restrictions at the time of the study) were required be followed at all times, such as hand-washing, avoiding touching the face, and social distancing by standing at least 2 m apart at all times. In order to establish group peership with their school, participants chose a logo and a mascot for their school team. This is a commonly used way of heightening group identification by making group identity salient in children, as demonstrated by previous developmental research (Nesdale and Dalton, 2011).

#### Ingroup Norm

Participants then received a message from their school team, which was randomized by the survey software Qualtrics, and so they received one of the following messages: Participants in the control condition were shown the following message: “*Welcome to the team. Our goal is to win this competition!*” Participants in the critical norm condition were shown the following message: “*Welcome to the team. Our goal is to win this competition! Now that you are a peer of this team, you should know what is important to us. We think that we should make sure something is true before we believe it, no matter who it comes from.”*

#### Group Membership

Next, participants were introduced to ingroup and outgroup peers who were also competing in the Spelling Bee competition. These group peers were always gender-matched to the participants.

Participants in the ingroup misinformer/outgroup target condition were first introduced to Sam, who was representing the same school as the participant, and was therefore in the participant’s ingroup. The participant was then shown other ingroup peers who were also representing the participant’s school in the competition, Charlie, Jamie, Joe (or Jo), and Jordan. Then, an outgroup peer, Alex, was introduced, who was representing the opposition school team in the competition.

Participants in the outgroup misinformer/ingroup target condition were first introduced to Alex, who was representing the same school as the participant, and was therefore in the participant’s ingroup. The participant was then shown other ingroup peers who were also representing the participant’s school in the competition, Charlie, Jamie, Joe (or Jo), and Jordan. Then, an outgroup peer, Sam, was introduced, who was representing the opposition school team in the competition.

After being shown their ingroup and outgroup peers, participants were then informed that on the final day of the National Interschool Spelling Bee Competition, Sam posted a video to WhatsApp. Alongside the video, Sam had written: “*Just saw Alex breaking social distancing rules!* [shocked emoji].”

Participants in the control condition saw responses by most of their fellow ingroup peers underneath Sam’s comment, which were all in support of their teammate, and so were congruent with the default expectations of a group – but these responses varied depending on the group identity of the misinformer. If participants were in the ingroup misinformer/outgroup target condition, their ingroup peers were showing their support for fellow ingroup peer Sam (“*I trust Sam, he/she is right. Alex was breaking the rules*!”). If participants were in the outgroup misinformer/ingroup target condition, their ingroup peers were disagreeing with outgroup peer Sam to support fellow ingroup peer Alex (“*I trust Alex, he/she can’t be breaking the rules. Sam is wrong*”). The ingroup peers within the WhatsApp group always defended the ingroup peer whether they were the misinformer or target to provide ecological validity to the context. This is because from an early age, ingroup peers typically expect loyalty from members of their peer group (Abrams and Rutland, 2008; Yazdi et al., 2020).

Participants in the critical norm condition saw responses by most of their fellow ingroup peers underneath Sam’s comment, which were always in support of seeing more information, and so congruent with the explicit norm those participants had seen in the beginning (“*I think we should wait for more information to see if this is true*”). They saw this response from fellow ingroup peers regardless of whether their misinformer was an ingroup or outgroup peer.

In all conditions, participants were then shown the reaction of a final teammate, Jordan, who despite being an ingroup peer, was deviating from the responses of fellow ingroup peers. In the control condition, Jordan said: “*I think we should wait for more information to see if this is true.”* In the critical norm condition, Jordan said: “*I trust Sam, he/she was breaking the rules. Alex was breaking the rules!*” when Sam was an ingroup misinformer and “*I trust Alex, he/she can’t be breaking the rules. Sam is wrong*” when Sam was an outgroup misinformer.

All participants were then informed that Sam’s video was misleading, and the angle from which it was taken did not convey the truth, which was that Alex was indeed social distancing. All participants saw the following message: ***BUT**…Sam’s video was taken from very far away, which made it look like Alex was not social distancing. Other videos and pictures, which were taken closer to the team, showed that **Alex was standing 2 m away** from everyone else. Sam did not check this information before posting the video to WhatsApp.*

### Measures

#### Judgment of Misinformer

Participants were asked to give their judgment of Sam, the misinformer, “*Sam, from [relevant group affiliation] posted the video to WhatsApp. How do you feel about Sam?”* They then selected their response from a 5-point scale showing faces ranging from 1 (very unhappy face) to 5 (very happy face).

#### Inclusion of Misinformer

Participants were then asked to decide whether they wanted Sam to be a part of their team, “*Do you want Sam to still be in/join your team?*” They selected their answer from a 5-point Likert scale which went from 1 (“Definitely not”) to 5 (“Definitely yes”).

#### Intentions of Misinformer

Participants were finally asked about the misinformer’s intentions, “*Do you think Sam thought he/she was doing something OK when he/she posted the video and comment on WhatsApp*?” They gave their responses on a 5-point Likert scale which went from 1 (“Definitely not”) to 5 (“Definitely yes”).

#### Reasoning Coding

After participants indicated their judgment and inclusion decisions about the Misinformer, they were asked each time to elaborate on their response by answering open-ended ‘Why?’ questions. These responses were coded in accordance with Social Domain Theory (Turiel, 1998; Smetana, 2013) and the three distinct domains of reasoning it outlines (moral, social-conventional, and psychological). Based on these domains, five subcategories were drawn which emerged the most from within the participants’ responses, having been referred to more than 10% of the time (see Table 1 for examples of each subcategory from the participants’ responses). The coding was conducted by two trained coders, one of whom was blind to the hypotheses of the study, on 25% of the sample of responses (*n* = 89). A high level of interrater agreement was achieved for both measures (Judgment of Misinformer: Cohen’s κ = 0.98; Inclusion of Misinformer: Cohen’s κ = 0.96). Participants who referenced moral concerns in relation to fairness and equality or personal concerns in terms of autonomy were dropped from the final analyses due to the frequency of their use being less than 10%, along with participants who referenced other matters (e.g., lack of information). Often, participants referenced more than one subcategory in the same response (e.g., “*Everyone in the team is important for us to win, and everyone makes mistakes”*) and so multiple coding was adopted. Each subcategory was given a code, where 1 = full use of the subcategory, 5 = even use with another subcategory, 0 = no use of the subcategory. Both negative and positive responses were included in each subcategory.

**TABLE 1:** Social-moral reasoning categories, with examples of participant responses for each of the subcategories which are in bold.

	Examples
1. Moral	
**Welfare** Concerns relating to harm including hurting feelings	“because she did something pretty mean”
**Lying and deceit** Any references to lying, deception, but also honesty	“he made one of our team mates look like he was breaking rules”
2. Social-conventional	
**Group functioning** References to winning, group dynamics and loyalty	“because he’s helping us and sounds like a good team mate”
**Conventional norms and expectations** Non-competitive references to rules and norms	“because if she has not broken the rules she deserves to [be included]”
3. Personal	
**Personal** References to personal choice, traits and perspective taking	“because he could have just made a mistake and misinterpreted Alex’s social distancing”

### Data Analytic Plan

The first analysis set out to examine H1, H2, and H3, which concerned the misinformer evaluations (judgments, inclusion and intentionality of the misinformer). These hypotheses were analyzed with a 2 (age group: children vs. adolescents) × 2 (ingroup norm: control condition vs. critical norm condition) × 2 (group membership: ingroup misinformer/outgroup target vs. outgroup misinformer/ingroup target) univariate ANOVA. Follow up independent-sample *t*-tests for interaction effects were conducted with Bonferroni corrections for multiple comparisons applied. Participants’ interest in spelling and their identification with their school group was controlled for in each analysis.

To investigate H4, another analysis was conducted on participants’ open-ended reasoning responses for Judgments and Inclusion of Misinformer. This was achieved with a 2 (age group: children vs. adolescents) × 2 (ingroup norm: control condition vs. critical norm condition) × 2 (group membership: ingroup misinformer/outgroup target vs. outgroup misinformer/ingroup target) × 5 (reasoning: welfare, lying and deceit, group functioning, conventional norms and expectations, personal) ANOVA with repeated measures on the final variable (for Inclusion of Misinformer, welfare was dropped as a moral subcategory as only 5% participants made references to such concerns). Where sphericity was violated, the Huynh–Feldt adjustment was reported. Pairwise comparisons were observed for main effects, and independent-samples *t*-tests were used to break down interactions. Participants’ interest in spelling and their identification with their school group was controlled for in each analysis.

## Results

### Judgment of Misinformer

As expected, there was a significant main effect of group membership on Judgment of Misinformer, *F*(1,343) = 16.02, *p* < 0.001. Participants with an ingroup misinformer/outgroup target judged Sam, the misinformer, more positively (*M* = 2.81, *SD* = 0.96) than those with an outgroup misinformer/ingroup target (*M* = 2.38, *SD* = 1.03). There were no significant main effects of ingroup norm (*p* = 0.58) on this measure, and no interactions.

### Inclusion of Misinformer

Again, as anticipated, there was a significant main effect of group membership on Inclusion of Misinformer, *F*(1,343) = 79.73, *p* < 0.001. Participants with an ingroup misinformer/outgroup target included Sam, the misinformer, more (*M* = 3.64, *SD* = 1.10) than those with an outgroup misinformer/ingroup target (*M* = 2.49, *SD* = 1.16). There were no significant main effects of ingroup norm (*p* = 0.36) on this measure, and no interactions.

### Intentionality of Misinformer

**FIGURE 1:**
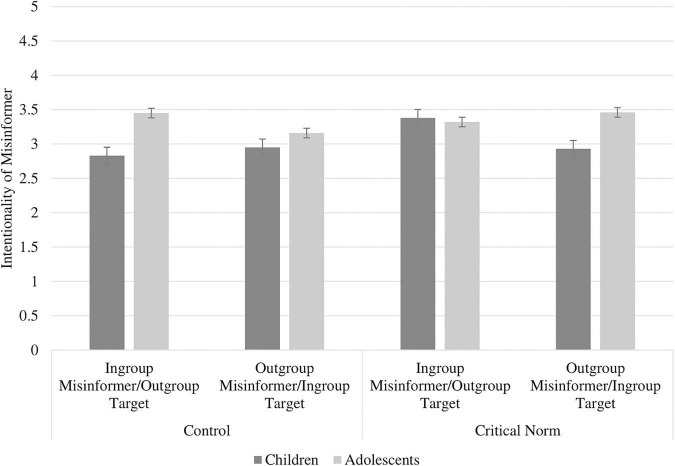
Participants’ intentionally attributions (1 = Definitely not OK intentions, 5 = Definitely OK intentions) of the misinformer by age group, ingroup norm, and group membership (with standard errors bars).

There were no significant main effects of either age group (*p* = 0.08), ingroup norm (*p* = 0.12) or group membership (*p* = 0.28) on Intentionality of Misinformer. However, there was a significant three-way interaction effect between age group, ingroup norm and group membership, *F*(1,343) = 4.70, *p* = 0.031 (see Figure 1). Age-related differences in intentionality attributions emerged in both norm type conditions, dependent on the identity of the misinformer/target.

Amongst participants in the control condition, there was a significant difference between children and adolescents with an ingroup misinformer/outgroup target *t*(76) = 2.45, *p* = 0.017, but not between children and adolescents with an outgroup misinformer/ingroup target (*p* = 0.32). Adolescents in the control condition attributed more positive intentions to an ingroup misinformer with an outgroup target (*M* = 3.43, *SD* = 0.82) than children in the control condition did (*M* = 2.83, *SD* = 1.17). In the control condition, children’s intentionality attributions did not significantly differ by group membership, (*p* = 0.61) nor did adolescents’ (*p* = 0.21).

Amongst participants in the critical norm condition, there was a significant difference between children and adolescents with an outgroup misinformer and an ingroup target *t*(71) = 2.16, *p* = 0.034, but not between children and adolescents with an ingroup misinformer and an outgroup target (*p* = 0.78). Adolescents in the critical norm condition attributed more positive intentions to an outgroup misinformer with an ingroup target (*M* = 3.46, *SD* = 0.92) than children in the critical norm condition did (*M* = 2.93, *SD* = 1.07). In the critical norm condition, children’s intentionality attributions did not significantly differ by group membership, (*p* = 0.055) nor did adolescents’ (*p* = 0.53).

Amongst the children in the sample, there was a significant difference in intentionality attributions by ingroup norm for those with an ingroup misinformer and outgroup target, *t*(101) = 2.33, *p* = 0.022, but not with an outgroup misinformer and ingroup target (*p* = 0.95). Children in the critical norm condition attributed more positive intentions to an ingroup misinformer with an outgroup target (*M* = 3.38, *SD* = 1.21), than children in the control condition did (*M* = 2.83, *SD* = 1.17).

Amongst the adolescents in the sample, there were no significant differences in intentionality attributions by ingroup norm for neither those who had an ingroup misinformer and an outgroup target (*p* = 0.60), nor those adolescents who had an outgroup misinformer and an ingroup target (*p* = 0.19).

Taken altogether, the three-way interaction shows that in the control condition, adolescents attributed more positive intentions to a misinformer compared with children, but only when the misinformer was an ingroup peer and their target was an outgroup peer. In the critical norm condition, adolescents also attributed more positive intentions to the misinformer, but only when the misinformer was an outgroup peer and their target was an ingroup peer. For children specifically, there was an effect of the ingroup norm manipulation, where being allocated to the critical norm condition resulted in more positive intentionality attributions toward the misinformer, but only when the misinformer was an ingroup peer and their target was an outgroup peer.

### Reasoning About Judgment of Misinformer

There was a significant reasoning by age group interaction, *F*(1,349) = 6.31, *p* = 0.012, η^2^ = 0.02 in participants’ open-ended responses about their Judgments of the Misinformer. Welfare reasoning (e.g., “*because he was trying to get Alex into trouble*”) was used more by children (*M* = 0.16, *SD* = 0.34) than adolescents, (*M* = 0.05, *SD* = 0.19), *t*(335) = 3.85, *p* < 0.001. Lying and deceit reasoning (e.g., “*because Sam could be making it up*”) was also used more by children (*M* = 0.23, *SD* = 0.40) than adolescents (*M* = 0.08, *SD* = 0.24), *t*(342) = 4.37, *p* < 0.001. However, reasoning referencing conventional norms and expectations (e.g., “*because she might of broken the rules*”) was prioritized by adolescents (*M* = 0.23, *SD* = 0.39) more than children (*M* = 0.09, *SD* = 0.25), *t*(231) = 3.83, *p* < 0.001. This interaction shows that children’s reasoning about their judgments of the misinformer included more moral concerns (i.e., welfare, lying and deceit), whereas adolescents included more social-conventional matters (i.e., conventional norms and expectations).

### Reasoning About Inclusion of Misinformer

There was also a significant reasoning by age group interaction, *F*(3,347) = 2.76, *p* = 0.046, η^2^ = 0.008 in participants’ open-ended responses about their Inclusion of Misinformer evaluation. Children used more lying and deceit reasoning (e.g., “*because I think she’s lying to us so that’s why I don’t want her to join our team*”) to justify their inclusion decisions (*M* = 0.24, *SD* = 0.41), than adolescents did (*M* = 0.09, *SD* = 0.26), *t*(346) = 4.46, *p* < 0.001. Adolescents used more group functioning reasoning (e.g., “*because she doesn’t seem like a team player”*) in their inclusion justifications (*M* = 0.33, *SD* = 0.45), than children did (*M* = 0.23, *SD* = 0.40), *t*(293) = 2.17, *p* = 0.016. These interaction shows that children’s reasoning about their inclusion evaluations of the misinformer, referred to more moral concerns (lying and deceit) whereas adolescents’ reasoning referred to social-conventional matters more (group functioning).

There was also a significant reasoning by group membership interaction, *F*(3,347) = 4.24, *p* = 0.007, η^2^ = 0.012. Participants cited lying and deceit concerns (e.g., “*that is because he is probably a liar*”) more when they had an outgroup misinformer and an ingroup target (*M* = 0.23, *SD* = 0.40), than when they had an ingroup misinformer and an outgroup target (*M* = 0.13, *SD* = 0.31), *t*(346) = 4.46, *p* < 0.001. Alternatively, participants used more personal reasoning, (e.g., “*because I think Sam thought that Alex wasn’t social distancing because it was far away*”) when they had an ingroup misinformer and an outgroup target (*M* = 0.24, *SD* = 0.40), than when they had an outgroup misinformer and an ingroup target (*M* = 0.11, *SD* = 0.30), *t*(335) = 3.45, *p* < 0.001. This shows that inclusion evaluations were justified using moral concerns such as lying and deceit more for an outgroup misinformer with an ingroup target, whereas inclusion evaluations about an ingroup misinformer with an outgroup target were justified more using personal reasoning.

## Discussion

The present study used a Social Reasoning Developmental (SRD; Rutland et al., 2010) approach to understand how children and adolescents evaluated and reasoned about an individual spreading misinformation, depending on factors such as the group membership of the misinformer and their target, as well as the norm of the participants’ ingroup. In terms of children’s and adolescents’ judgment of the misinformer, and their decision to include the misinformer in their group, results showed that only group membership impacted responses. As predicted in H1, when the misinformer was an ingroup peer who had spread misinformation about an outgroup peer, participants were more likely to be happy with and include the misinformer in their group. However, unlike moral evaluations, intentionality attributions were not directly affected by the group membership manipulation.

With regards to the ingroup norm, contrary to predictions (H2), there was no main effect of the critical norm condition on participants’ moral evaluations of the misinformer. In terms of intentionality attributions, while there were no main effects of either age group or misinformer identity on participants’ attribution of intentions, there was a three-way interaction between age group, ingroup norm and group membership. The three-way interaction indicated developmental effects in line with predictions (H3), insofar as adolescents attributed more positive intentions to the misinformer compared with children in both norm manipulation conditions, but this was dependent on the identity of the misinformer/target. The interaction also highlighted the role of the critical norm condition in increasing children’s intentionality attributions compared to the control condition, but only when the misinformer was an ingroup peer who had spread misinformation about an outgroup peer. Finally, H4 was also supported as children used more moral reasoning (welfare, lying, and deceit) to justify their evaluations of the misinformer than adolescents did, whereas adolescents comparatively used more social-conventional (group functioning, conventional norms, and expectations) reasoning than children did.

### The Importance of Group Identity

In line with Social Identity Theory (SIT; Tajfel and Turner, 1986), children and adolescents did indeed exhibit an ingroup bias when morally evaluating a misinformer. This study is the first of its kind as it focused on children’s and adolescents’ moral evaluations of the individual spreading misinformation in an intergroup context, and so provides a novel insight into a phenomenon that is becoming increasingly more relevant to our current digital society. This finding is important as it implies that from the age of 8, children differentiate between individuals from their group and individuals from a different group even when they commit the same morally dubious act – and they continue to do so through to adolescence. Crucially, having an ingroup norm that emphasized being questioning of information, and thus an ingroup peer who spread information without checking, did not have a direct impact on participants’ responses. This could have been because of the failure of the critical norm manipulation itself, which due to the strong ingroup bias demonstrated in this context, may have required more emphasis and frequent reminders in order have an impact. As critical thinking skills training can be effective in reducing belief in misinformation, it is possible that for a critical norm to be effective in impacting *moral* evaluations *of* the misinformer, it would need to be prioritized over the strong ingroup preference that children display. The question is then what may encourage children and adolescence to prioritize being critical over supporting their ingroup.

From the trends observed in participants’ reasoning, it is clear that the group identity of the misinformer and their target led to different justifications being used by the participants. When participants had an outgroup misinformer and an ingroup target, they cited lying and deceit concerns to justify their inclusion evaluations, which involved more accusations of dishonesty. In comparison, participants with an ingroup misinformer and an outgroup target used more personal reasoning to justify their inclusion evaluations, which tended to include more perspective taking on the misinformer’s part. This highlights that despite the misinformer’s claim being the same for participants in both misinformer identity conditions, having an ingroup peer as a misinformer can result in more likelihood to engage in mental state understanding, whereas having an outgroup peer as a misinformer results in less belief in their claim. This is in line with past research showing that children and adolescents are more likely to consider the mental states of ingroup peers than outgroup peers (McLoughlin and Over, 2017; Gönültaş et al., 2020). It is also an important insight, for it suggests that to counteract the ingroup bias in accepting misinformers, emphasizing concerns in these particular areas of the moral and social domains of reasoning may be most effective.

### Developmental Differences

There were important age-related differences that emerged in the present study. Firstly, children made more references to moral concerns, such as welfare and lying and deceit, than adolescents did when judging the misinformer. On the other hand, adolescents’ reasoning about their judgments consisted of more references to social-conventional matters, such as group functioning and conventional norms and expectations. This developmental shift is congruent with previous research, which claims that children are relatively more concerned with moral factors in their reasoning, whereas adolescents become comparatively more concerned with social-conventional or psychological matters (Killen and Rutland, 2011; Killen et al., 2013) as explained by adolescents’ more fervent interest in group norms and dynamics (Rutland et al., 2015).

Furthermore, as predicted, and in line with previous research (Killen et al., 2011; Jambon and Smetana, 2013), participants’ intentionality attributions became more positive with age; adolescents made more positive intentionality attributions about the misinformer compared with children. This age trend, when broken down, was partially due to the two conditions in which adolescents were more likely than children to believe a misinformer was spreading misinformation unintentionally.

First, in the control condition where loyalty to the group was expected, adolescents attributed significantly more positive intentions to an ingroup misinformer compared with children. This could have been due to adolescents’ superior perspective taking abilities, which may have resulted in them being more likely to regard the misinformer’s actions as accidental, an effect exacerbated by the ingroup status of the misinformer, the outgroup status of the target and the expectations of loyalty in the group.

Second, the other condition that showed an age difference was when there was an ingroup norm that encourages thinking critically. In this condition, children’s intentionality attributions for an outgroup misinformer were significantly more negative than adolescents’, suggesting the critical norm facilitated a dislike for the outgroup amongst the children only. This could have been linked to the evidence that shows younger children are worse at acknowledging and interpreting the mental states of outgroup peers, and consider the mental states of ingroup peers more, than older children and adolescents do (McLoughlin and Over, 2017; Gönültaş et al., 2020).

Further, when children were assigned to the critical ingroup norm condition, they made more positive intentionality attributions about an ingroup misinformer than when they were in the control condition. This finding opens up the possibility that a group norm of being critical may encourage children to be more positive in their perspective taking regarding an ingroup misinformer’s intentions. Hence, a norm of thinking critically may have made children question their assumption of a misinformer’s intentions, but in a negative direction for outgroup peers and in a positive direction for ingroup peers, given children are better at interpreting their ingroup’s mental states. The present study did not take an isolated measure of participants’ social-cognitive perspective taking ability, such as their Theory of Mind ability, so it is not possible to underpin the mechanism responsible for this effect. Future research should, therefore, explore Theory of Mind ability in relation to age-related effects of a critical ingroup norm on intentionality attribution.

These developmental differences may also have been linked to children’s open-ended justifications of both moral evaluations, which were significantly more concerned with lying and deceit than adolescents. Hence, it is possible that given the misinformer’s intentions were ambiguous in the study, children were more likely to presume the misinformer had deliberately spread misinformation, and so committed an intentional act of deception. This is supported by prior research, which has shown that even when a false claim is made unintentionally, children justify negative evaluations of the claim with references to lying (Rizzo et al., 2019). Developmental differences in intentionality attributions, therefore, are necessary to consider in the context of misinformers, especially as much of misinformation online can be spread unintentionally or with ambiguous intentions. These developmental differences in attributing intentions should, therefore, be considered when designing interventions that combat the spread of misinformation, as age may play a key role in whether someone is perceived as being intentionally or unintentionally deceptive.

### Limitations and Future Directions

The manipulation of the ingroup norm, which failed to have a predicted effect, is a limitation of the study. This may have been because, with regards to the critical norm condition, a norm message from their school team telling them what they value may not have been enough to influence their judgments and decisions. Rather, seeing their fellow peer group members being critical, or being presented with a context that explicitly highlights moral concerns over group concerns might have been needed. Without seeing what being critical means in practice, it is possible that it is not strong enough to influence the participants as much as the manipulation of the misinformer’s identity did. Given this was the first time a norm of this kind was used in a study manipulation, it is possible that with more development and further studies, it can become more effective. The control condition may also have been a possible weakness for not having an explicit norm, and for assuming loyalty to be the norm without making it a more salient expectation. Together, this may have been why the effect of the norm was not as predicted, and may need to be strengthened in future studies. From this study alone, the findings suggest that critical thinking is something that requires more than just a single norm message to encourage in children and adolescents, and perhaps first it needs to combat the strong effect of ingroup biases.

The present study’s design manipulated the group membership of both the misinformer and the target of the misinformation, so the misinformer was always in the opposing team of their target. This was done to create an intergroup context with ecological validity, as in real-life contexts, it is unlikely for a group member to share a false accusation about their fellow group member in a competitive intergroup context, resulting in a disadvantage for their own group. We still acknowledge that not having comparative groups where an ingroup misinformer targets a fellow ingroup peer, and an outgroup misinformer targets a fellow outgroup peer, is a limitation of the present study. Adding such manipulation conditions would have made the conclusions much clearer in terms of whether it is the group membership of the misinformer or the target that drives the evaluations made by our sample, which we are aware is currently unclear from our present study. Future research should include such comparisons for more comprehensive conclusions and include evaluation questions about both the misinformer and their target.

Ingroup and outgroup membership in this study was related to school teams in a spelling competition. We recognize that the inclusion evaluation may have been affected by the presumption that an individual cannot easily leave their school team to join another. Nonetheless we would have anticipated similar results in an alternative intergroup context other than a school, such as a sports team, where moving between groups is also potentially difficult and risky. In future research, this question could be reframed around the inclusion of the misinformer in a future event and a different intergroup context, for instance, rather than the same inter-school context.

It should also be noted that this research was conducted during the COVID-19 pandemic, in-between national lockdown cycles and during strong, government-led norms about social distancing and following rules for the sake of saving lives. It is unclear from this study how the COVID-19 social distancing guidelines, which feature in the intergroup context, were perceived by the participants. This was not a factor we had controlled for, as to our knowledge, there have not been any studies conducted around children and adolescents’ reasoning about COVID-19 rules. It is therefore uncertain whether they perceived social distancing as a matter of moral concern, social-convention or personal choice. This lack of certainty is a limitation of the present study, but a definite exploration for future research to undertake.

Nonetheless, in some cases participants’ reasoning about their evaluations indicated that the welfare concerns of not social distancing (such as spreading of the virus, getting sick, etc.) were not a priority. From the whole sample, welfare concerns in the context of *health risk* was only commented on twice (e.g., “*broke rules risking people’s health*” and “*because he is telling her friend to keep 2 m so she doesn’t get COVID-19*”), suggesting COVID-19 and its guidelines were not seen as much of a moral matter, but was frequently commented on in the context of *just* following the social distancing rules (e.g., “*because she does not break the social distancing rules*”), which on its own was regarded as a social-conventional matter. While these indications alone are insufficient to draw conclusions from, there is a likelihood that the participants of the study viewed the social distancing guidelines as a social-conventional matter rather than a moral welfare concern.

Overall, the findings in this study make a strong case for focusing on group identity effects, as highlighted by both evaluation responses and open-ended reasoning, when trying to address ingroup biases toward misinformers. In the adult literature, it has been demonstrated that misinformation belief and spread can be attributed to individuals’ desire to gain the acceptance of an identity group, often at the expense of maintaining the accuracy of the information itself (Levy et al., 2021). The present study and its findings arguably tap into a similar mechanism occurring in childhood and adolescence, where participants who share a group identity with the misinformer make more favorable evaluations of the misinformer, even attempting to understand their perspective more. The developmental differences in intentionality attribution and choice of reasoning also highlight that evaluations of misinformers are subject to age-related influences. It is therefore crucial that interventions that focus on challenging children and adolescents’ susceptibility to misinformation and its source should consider the intergroup factors that may be at play, alongside emerging developmental differences. For this reason, the present study makes an important and necessary Contribution To The Field of understanding children and adolescents’ evaluations of peers who spread misinformation about fellow peers.

### Conclusion

The present study provided a novel contribution to existing research by demonstrating that group membership and age-related differences influence moral and intentionality evaluations and reasoning about a misinformer. Ingroup preferences emerged both when participants morally evaluated the misinformer, and when they justified those responses. Participants were more likely to engage in perspective taking when the misinformer was an ingroup peer targeting an outgroup peer, and more likely to level an accusation of dishonesty toward an outgroup misinformer targeting an ingroup peer. The age-related differences highlighted in the present study also extend previous research, reinforcing the relative importance of moral concerns for children and social-conventional matters for adolescents, and providing insights from children’s reasoning for their more negative intentionality attributions compared with adolescents. Altogether, this study argues for an approach to tackling the spread of misinformation that takes into account factors such as shared group identity with, and developmental differences in attribution of intentionality to the spreaders of misinformation itself.

## Data Availability Statement

The raw data supporting the conclusions of this article will be made available by the authors, without undue reservation.

## Ethics Statement

The studies involving human participants were reviewed and approved by Dr. Nick Moberly, University of Exeter Ethics Committee. Written informed consent to participate in this study was provided by the participants’ legal guardian/next of kin.

## Author Contributions

AF, AR, and AA conceived of the study and contributed to manuscript revision, read, and approved the submitted version. AF carried out the data collection, performed the data analysis, with assistance from EK on reliability coding, and wrote the first draft of the manuscript. All authors contributed to the article and approved the submitted version.

## Conflict of Interest

The authors declare that the research was conducted in the absence of any commercial or financial relationships that could be construed as a potential conflict of interest.

## Publisher’s Note

All claims expressed in this article are solely those of the authors and do not necessarily represent those of their affiliated organizations, or those of the publisher, the editors and the reviewers. Any product that may be evaluated in this article, or claim that may be made by its manufacturer, is not guaranteed or endorsed by the publisher.
